# Homogenization and thermal processing reduce the concentration of extracellular vesicles in bovine milk

**DOI:** 10.1002/fsn3.3749

**Published:** 2023-10-08

**Authors:** Anna P. Colella, Anuradha Prakash, John J. Miklavcic

**Affiliations:** ^1^ Schmid College of Science and Technology Chapman University Orange California USA; ^2^ School of Pharmacy Chapman University Irvine California USA

**Keywords:** bovine milk, extracellular vesicles, homogenization, pasteurization

## Abstract

Extracellular vesicles (EVs) in bovine milk confer beneficial physiologic effects to consumers. Industrial processing treatments may affect the amount or bioactivity of EVs intrinsic to bovine milk. We investigated how the content and concentration of EVs were affected by homogenization and thermal processing of raw bovine milk. Raw milk was processed by homogenization, low‐temperature (LT) heat, or pasteurization [high‐temperature short‐time (HTST) and ultra‐high‐temperature (UHT)] in a pilot processing facility. EVs were isolated from the raw and processed bovine milk using differential ultracentrifugation and quantified using a nanoparticle tracking analyzer. Bovine milk EVs were assessed for total miRNA and protein concentrations standardized to particle count using a fluorometric assay. There were 1.01 × 10^10^ (±3.30 × 10^9^) EV particles per ml of bovine milk. All industrial processing treatments caused >60% decrease in EV concentration compared to the raw bovine milk. Homogenization and heat treatments independently and additively reduced the content of EVs in bovine milk. The averages of total miRNA/particle and total protein/particle concentrations were elevated threefold by low‐temperature heat‐processing treatment relative to HTST and UHT pasteurizations. The average diameter of EVs was reduced by 11%–16% by low temperature compared to raw milk (127 ± 13 nm). Homogenization and pasteurization indiscriminately reduce the EV concentration of bovine milk. Smaller EVs with higher protein content resist degradation when processing bovine milk at sub‐pasteurization temperature. This new foundational knowledge may contribute to food product development on the preservation of EVs in processed dairy products, including bovine milk‐based infant formulas that some newborns are dependent on for adequate growth and development.

## Graphical abstract

Processing may affect the amount or bioactivity of EVs intrinsic to bovine milk. Homogenization and pasteurization reduced the concentration of EVs in bovine milk. Higher protein content conferred resilience of EVs to sub‐pasteurization temperature.

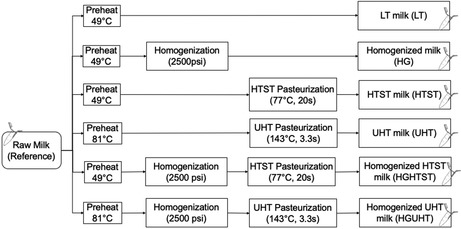

## 1 INTRODUCTION

There is a growing body of evidence that bovine milk EVs regulate physiologic processes that support human growth and homeostasis. EVs from bovine milk are purported to influence wound healing (Kim et al., 2022), promote bone health, and protect intestinal integrity. EVs from bovine milk promoted the proliferation of osteogenic Saos‐2 cells (Go et al., 2021), and reduced the expression of enzymes that destroy cartilage in an ex vivo study of human cartilage explants (Pieters et al., 2022). In a tissue culture model of intestinal epithelium, bovine milk EVs prevented interferon‐gamma and lipopolysaccharide induced increases in proinflammatory cytokines interleukin (IL)‐17, tumor necrosis factor‐alpha, IL‐6, and IL‐1 beta (Mecocci et al., 2022). In addition, dextran sulfate sodium‐induced increases in IL‐6 and tumor necrosis factor‐alpha were abrogated in mice treated with bovine milk EVs (Du et al., 2022). Whether EVs remain intact and retain biologic activity after processing of bovine milk is unknown. The purpose of this study was to evaluate the effects of homogenization and thermal processing on the content and composition of EVs in bovine milk.

Pasteurization and homogenization are common treatments applied to milk to ensure destruction of potential microbial pathogens and extend product shelf‐life of cheese, butter, and milk powder (van Asselt et al., 2017). Milk is typically preheated before homogenization to liquify the fat, decrease viscosity, and optimize homogenization outcomes (TetraPak, 2015). Homogenization (1450–3625 psi) is performed to reduce the size of fat globules and prevent the formation of a cream layer during storage. High‐temperature short‐time (HTST; 72°C for 15 s) and ultra‐high‐temperature (UHT; 138°C for 2 s) are the most common pasteurization parameters. These processing steps can also impact the functional and nutritional components of milk, including vitamin content and protein digestibility. Understanding the impacts of processing on EVs can inform food processing and safety standards to preserve nutritional bioactives in dairy products.

The miRNA and mRNA in bovine milk may be resistant to industrial manufacturing processes due to encapsulation in EVs (Izumi et al., 2012). The membrane bilayer of EVs confers some protection to the particles, as no difference was found in size or particle concentration of EVs isolated from semi‐skimmed milk after boiling (15 min, 105°C) and freezing in liquid nitrogen (Pieters et al., 2015). However, boiling and UHT treatments reduced the miRNA content in bovine milk (Kirchner et al., 2016). Pasteurized, store‐bought whole milk has only one‐third of the miRNA found in raw milk. These and other studies (Howard et al., 2015; Kleinjan et al., 2021) have assessed EVs in store‐bought processed milk. Assessing how processing affects the content and composition of EVs relative to a reference within the same batch of milk may allow for enhanced understanding of how EVs are affected by commercial processing.

Unless intentionally added, commercial infant formulas do not contain miRNA and mRNA in a quantity to sufficiently impact the growth and development of infants (Izumi et al., 2012; Melnik & Schmitz, 2017). Processed yogurts have decreased concentrations of miRNA when compared to pasteurized whole milk, possibly resulting from the activity of RNases that are produced by fermentation microbes. Homogenization decreases miRNA content in bovine milk by 50%, likely due to shear forces that compromise nanovesicle structure and protection of oligonucleotides (Howard et al., 2015). Challenges have also been cited in the analysis of macromolecule quantification and characterization from processed milk as isolation of EVs from pasteurized and homogenized milk results in co‐isolation of non‐EV protein, lipid, and RNA (Hansen et al., 2022). Importantly, miRNAs with putative targets are still abundant in appreciable quantity in homogenized and heat‐treated milk EVs (Shome et al., 2021).

Understanding the impacts of processing on EV content and composition may narrow the gap in health outcomes between babies fed human milk and bovine milk‐based infant formula. Findings from this study may be used to ensure that newborns reliant on formulas can obtain bioactive compounds initially present in bovine milk.

## 2 MATERIALS AND METHODS

This research was exempt from review [Chapman University Institutional Review Board (IRB‐21‐33)]. We have submitted all relevant data from our experiments to the EV‐TRACK knowledgebase (EV‐TRACK ID: EV230962, EV‐METRIC = 67%) (EV‐TRACK Consortium, et al., 2017).

### 2.1 Study design

**FIGURE 1:**
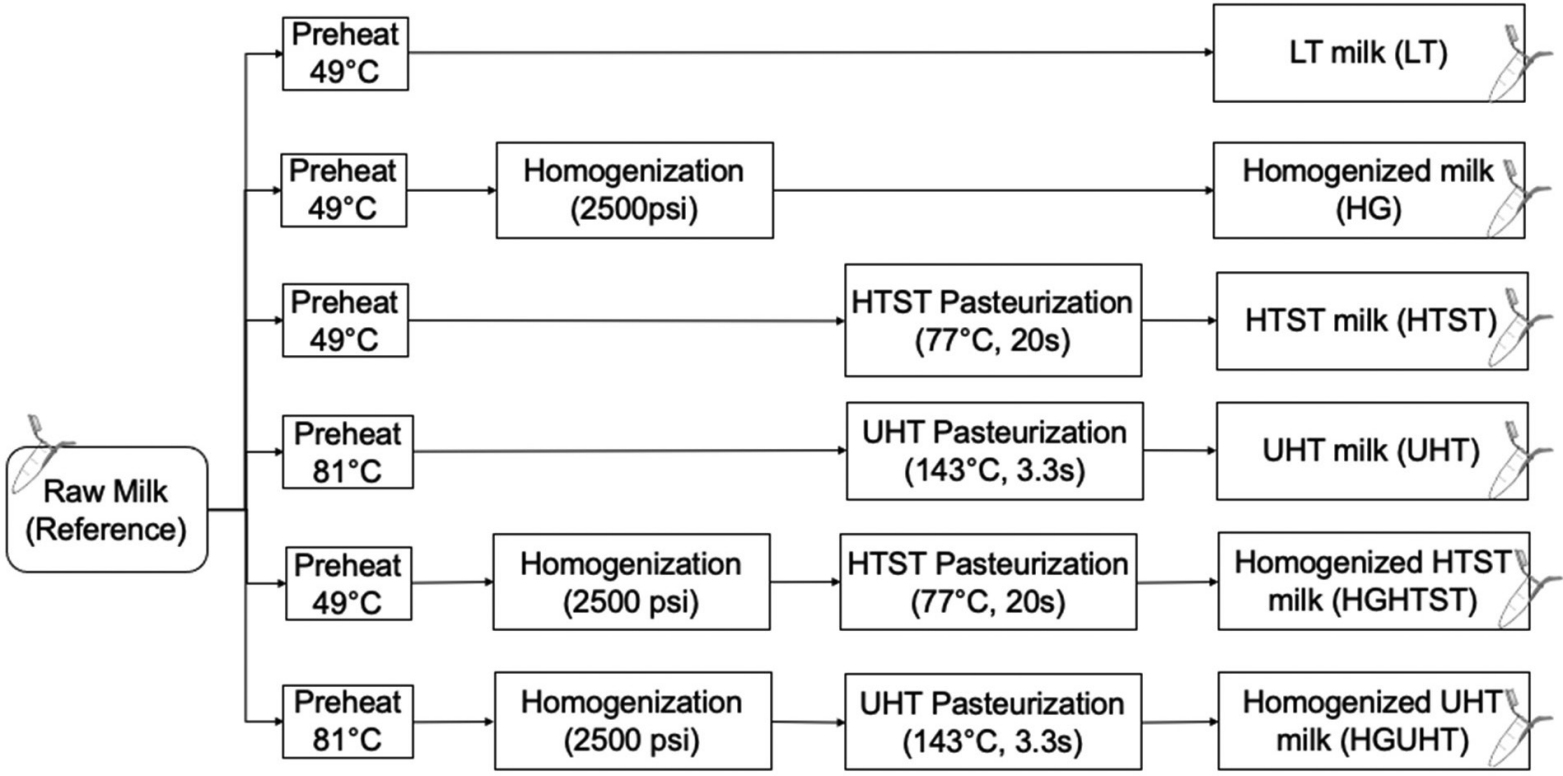
Processing flowchart. Extracellular vesicles were isolated from the six treatment groups and from the reference raw milk.

Two batches (M1 and M2) of pooled raw bovine milk were obtained from Stremicks Heritage Foods™ (Santa Ana, CA). The M1 batch was received at the dairy facility >12 h before collection by the researchers, and the M2 batch was collected <6 h after milking. A Milkoscan (Foss A/S, Hillerod, Denmark) was used to assess % fat, protein, lactose, total solids, and solids‐not‐fat. Within 24 h of milking, the raw milk was transported in sealed bags placed on ice and stored (4°C) at Chapman University (Orange, CA). The processing treatments mimicked unit operations commonly used in the commercial processing of raw milk. Six treatments each representing an experimental group were performed (Figure 1): LT, homogenization, HTST, homogenized high‐temperature short‐time (HGHTST), UHT, and homogenized ultra‐high‐temperature (HGUHT).

### 2.2 Homogenization and pasteurization

Pasteurization was conducted using a Microthermics Pasteurizer (Model LAB‐25 EHVH, Raleigh, NC, USA). Pasteurization was conducted under two conditions: HTST at 77°C for 20 s with a preheat temperature of 49°C, and UHT at 143°C for 3.3 s with a preheat temperature of 81°C. The duration and temperature of each treatment follow common practices utilized in the industry and are in accordance with 21CFR131.3 (FDA, 2022a). For treatments that included homogenization (HGHTST and HGUHT), an in‐line two‐stage GEA homogenizer (Model Ariete NS2006, Düsseldorf, Germany) was engaged (first stage 2000 psi; second stage 500 psi) in conjunction with the Microthermics pasteurizer. Homogenization occurred after the bovine milk was preheated to 49°C (TetraPak, 2015) at 2500 psi. In order to isolate the effects of homogenization from the effect of the mild heat treatment in preheating, the cluster of analysis LT was performed by treating milk at 49°C for 20 s and mimicking the preheating step of homogenization.

### 2.3 Validation of homogenization and pasteurization

Homogenization was validated using a light scattering instrument (Coulter, LS 230, Beckman Coulter, Atlanta, GA, USA) to measure volume‐average droplet diameters. The homogenized milk was frozen (−20°C) and stored for <3 days before subsequent analysis. Samples were thawed (37°C) and vortexed before measurements. Distilled water was used as a background solvent. The refractive indices used for milk fat were 1.46 and 1.33 for the aqueous continuous phase (Ransmark et al., 2019). Pasteurization was validated using Phosphatesmo MI test strips (Macherey‐Nagel, Düren, Germany) for the determination of alkaline phosphatase (ALP) in milk, according to manufacturer's instructions. A yellow color indicates the presence of functional ALP and a white color indicates inactivation of ALP. Measurements were taken in triplicate.

### 2.4 Extracellular vesicle isolation

After industrial processing treatment, the isolation of EVs was performed using a benchtop centrifuge (Sorvall Legend X1R, Thermo Fisher Scientific) equipped with a fixed‐angle rotor (F13‐14x50cy, Thermo Fisher Scientific) for speeds below 100,000 *g*, and an ultracentrifuge (Optima XE‐90, Beckman Coulter) equipped with a fixed‐angle rotor (Type 50.2 Ti, Beckman Coulter) for speeds at or above 100,000 *g*. Instrument acceleration and deceleration were set to maximum.

A differential centrifugation protocol was adapted from published protocols (Li et al., 2019; Munagala et al., 2016) and used for the isolation of EVs. To minimize the loss of EVs in freezing (Zonneveld et al., 2014), milk (90 mL) was defatted (2000 *g* for 10 min at 4°C, then 12,000 *g* for 40 min at 4°C), filtered through a cheesecloth (06‐665‐29, Thermo Fisher Scientific), and stored (−80°C) within 72 h of receipt. Defatted milk was thawed (12 h, 4°C), the supernatant was separated from debris after centrifugation (5000 *g*, 30 min, 4°C), and further resolved (10,000 *g*, 30 min, 4°C). Casein micelles were removed by ultracentrifugation (UC, 100,000 *g*, 60 min, 4°C), and the EV‐containing pellet obtained after UC (130,000 *g*, 60 min, 4°C) was resuspended in PBS (3 mL). Aliquots (1 mL) of EVs were transferred into microtubes and stored (4°C). Downstream analyses were performed within 72 h of EV isolation.

In the isolation procedure, non‐homogenized samples had a clear supernatant, while homogenized samples were turbid (Figure S1; Miklavcic et al., 2022). Since turbidity visually affected recovery, only a fraction of the entire EV‐containing supernatant was recovered. Volume losses during the isolation procedure were estimated by a composite index of weight of supernatant removed from UC tubes, and visual estimation of volume losses was assessed independently by two investigators and then agreed upon by consensus. Non‐homogenized groups including the reference raw milk group were standardized to a 5% loss coefficient, and all homogenized samples were standardized to a 15% loss coefficient in order to account for a volume of EV‐containing milk supernatant discarded during isolation.

### 2.5 Extracellular vesicle characterization

Global characterization was conducted using semiquantitative immunoblot (Exo‐Check, System Biosciences) for the identification of typically positive ([Flotillin‐1 [FLOT1], ICAM, CD81, CD63, ALG‐2‐interacting Protein X [ALIX], Annexin‐5 [ANXA5], and tumor susceptibility gene 101 [TSG10]) and negative (GM130 and EpCAM) markers according to manufacturer instructions. The membrane was developed using Clarity Max Western ECL Substrate (Bio‐Rad) according to manufacturer instructions and imaged with a Bio‐Rad ChemiDoc Imaging System equipped with a Bio‐Rad Image Lab Touch Software Version 2.2.0.08 (Bio‐Rad) using the chemiluminescence application.

Single vesicle characterization was conducted using scanning electron microscopy (SEM). SEM was conducted within 24 h of EV isolation using a Zeiss Gemini Sigma 300 (Zeiss) for EV visualization. The isolated EVs were resuspended in PBS and serial diluted in distilled water. SEM slides were prepared with 25 μL of dilutions and air dried for 12 h in sterile petri plates. Samples were coated with a thin layer of gold/palladium (Au/Pd 80/20%, 99.99% Au/Pd) using a Mini Sputter Coater/Glow Discharge System SC7620 (Quorum Tech).

Nanoparticle Tracking Analysis (NTA, ZetaView PMX120) and ZetaView Software Version 8.05.12 SP1 (Particle Metrix GmbH) were used to assess size and quantify EVs. The EVs resuspended in PBS were serially diluted in PBS in order to achieve 100–250 particles per frame to optimize instrument sensitivity. The NTA cell was rinsed with PBS after each reading. A dilution (1:250,000) of polystyrene (100 nm) beads was prepared to perform an autoalignment routine on the instrument. The post‐acquisition settings were set to a minimal brightness of 20, a maximum area of 1000 pixels, a minimum area of 20 pixels, a trace length of 15 frames, 5 nm/class, and 64 classes/decade. The camera control was set to a sensitivity of 75, a frame rate of 30 frames per second, and a shutter speed of 100. Two technical replicates were taken for each sample. The instrument was set to read from 11 lanes in the cell with two cycle reads per position. A minimum of eight valid positions were required to accept the data. Grubbs test was performed for outlier analysis of lanes, and outliers detected were removed from statistical analyses.

The Qubit miRNA Assay Kit (Thermo Fisher Scientific) was utilized to assess the concentration of miRNA of EVs using the Qubit 4 Fluorometer (Thermo Fisher Scientific) according to manufacturer instructions. Protein concentration of EVs was assessed with a Qubit 4 Fluorometer (Thermo Fisher Scientific) according to manufacturer instructions. For each analysis, three dilutions were prepared for each batch, and measurements were taken in triplicate.

### 2.6 Statistical analysis

Statistical analyses were conducted using RStudio (Version 1.2.1335, RStudio Inc.). Particle count and miRNA and protein concentrations were assessed in EVs and compared across the experimental groups. When the data obtained did not satisfy requirements for parametric ANOVA analyses due to heteroskedasticity, non‐parametric statistical testing (Kruskal–Wallis) was used. Dunn's test for comparison of multiple means was conducted post hoc. When the effects of homogenization and heating were assessed independently, the Mann–Whitney–Wilcoxon test was used for means comparisons. An *α* = .05 was used to designate statistical significance for hypothesis testing. All results are reported as mean ± SD.

## 3 RESULTS

### 3.1 Validation of homogenization and pasteurization

Proximate analysis of raw bovine milk is detailed (Table S1; Miklavcic et al., 2022) and in agreement with 21CFR131.110, wherein milk for human consumption should not have a fat content lower than 3.25% and solids‐not‐fat lower than 8.25% (FDA, 2022b). The size distribution of fat globules from homogenized milks (HG, HGHTST, and HGUHT) was characteristically lower than the reference (Figure S2; Miklavcic et al., 2022), indicating successful homogenization. The reference and LT groups were positive for ALP testing, while HTST, HGHTST, UHT, and HGUHT groups showed inactivation of ALP, indicating successful pasteurization (Figure S3; Miklavcic et al., 2022).

### 3.2 Global and single vesicle characterization

Semi‐quantitative immunoblot showed that the GM130 and EpCAM markers were negative and the FLOT1, ICAM, ALIX, CD81, CD63, ANXA5, and TSG101 markers were positive in EVs isolated from bovine milk (Figure S4; Miklavcic et al., 2022). Broad‐field and close‐up high‐resolution imaging of EVs from raw bovine milk using SEM displayed particles with a diameter of less than 200 nm (Figure S5; Miklavcic et al., 2022).

### 3.3 Extracellular vesicle quantification and size

**FIGURE 2:**
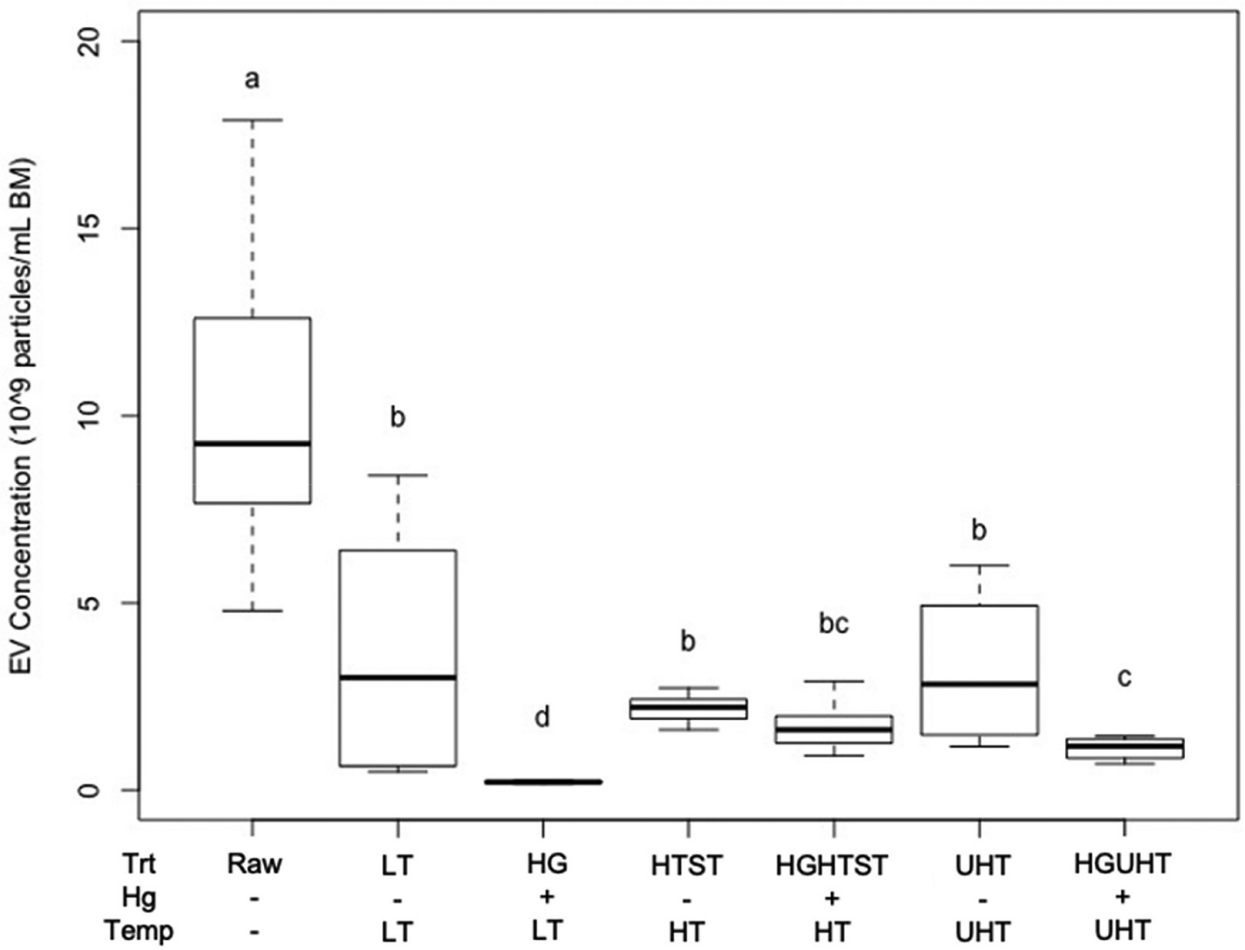
Boxplot of bovine milk extracellular vesicle (EV) concentration across processing treatments. Homogenization (HG) resulted in the greatest loss of EVs across treatments. HG and homogenized ultra‐high‐temperature (HGUHT) significantly decreased EV concentration compared to low‐temperature (LT) and ultra‐high‐temperature (UHT), respectively. Homogenized high‐temperature short‐time (HGHTST) did not significantly reduce the bovine milk's EV concentration compared to high‐temperature short‐time (HTST). Medians were compared using Kruskal–Wallis. Different superscript letters indicate significant differences (*n* = 44; *α* = .05). BM, bovine milk.

**FIGURE 3:**
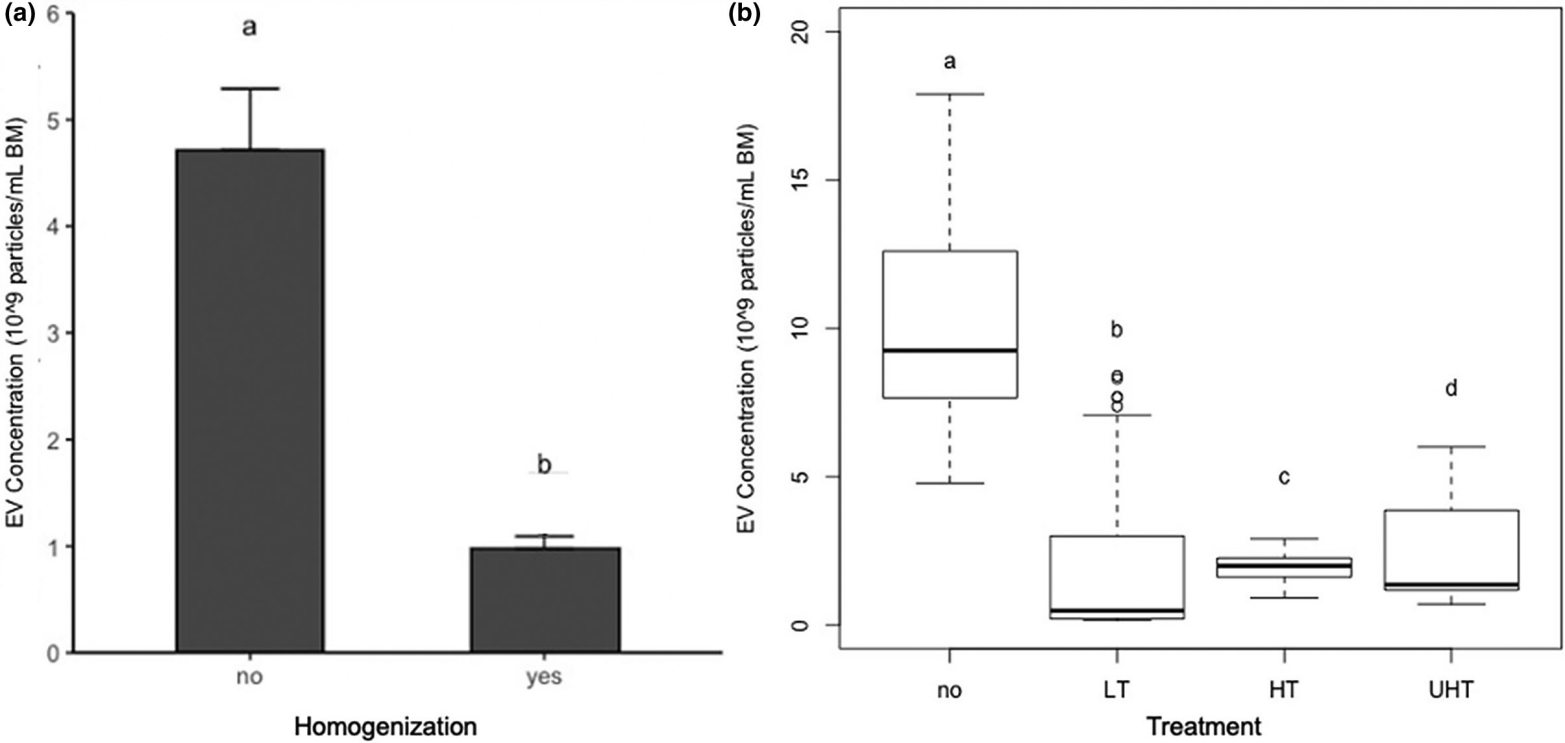
(a) Bar plot of extracellular vesicle (EV) concentration in bovine milk in combined non‐homogenized (low‐temperature (LT), high‐temperature short‐time (HTST), and ultra‐high‐temperature (UHT)) and homogenized milk (homogenization (HG), homogenized high‐temperature short‐time [HGHTST], and homogenized ultra‐high‐temperature [HGUHT]). Homogenization significantly reduced the concentration of bovine milk EVs from non‐homogenized samples. Mann–Whitney–Wilcoxon test was used to compare non‐homogenized to homogenized treatment groups. Bars depict the mean, and error bars represent the standard error of the mean. (b) Boxplot of bovine milk's EV concentration across heat treatments, combining non‐homogenized and homogenized samples. All heat treatments significantly reduced the concentration of EVs when compared to the reference raw milk. Medians were compared using Kruskal–Wallis. Different superscript letters indicate significant differences (*α* = .05). BM, bovine milk.

EV concentration and diameter measures across milk treatment groups are summarized (Table 1). The concentration of EVs in the reference was significantly higher than in each of the six treatment groups (Figure 2). Homogenization and heat treatments reduced EV concentration in an additive manner (*p* < .01). The concentration of EVs was reduced (*p* < .01) by homogenization compared to non‐homogenized samples independent of heat treatment (Figure 3a). Similarly, the concentration of EVs was reduced (*p* < .01) by all heat treatments, independent of homogenization, and was reduced most in the LT group compared to raw milk (Figure 3b). The average diameter of EVs was greater in the reference than in the LT‐treated groups (*p* < .05). The mean but not the median diameter was higher in EVs from the HGUHT group compared to the reference.

**TABLE 1:** Measurements of EVs from bovine milk across processing treatments.

Treatment	Homogenized?	Heat treatment?	Concentration (particle/mL)	Diameter (nm)	Unadjusted [protein] (μg/mL)	Protein (μg)/particle	Unadjusted [miRNA] (ng/mL)	miRNA (ng)/particle
Reference	No	No	1.01 × 10^10a^ ± 3.30 × 10^9^	127.02^a^ ± 12.91	390.55^a^ ± 32.13	4.02 × 10^−8a^ ± 1.01 × 10^−8^	484.61^a^ ± 16.78	5.16 × 10^−8a^ ± 1.34 × 10^−8^
LT	No	LT	3.60 × 10^9b^ ± 3.06 × 10^9^	106.21^b^ ± 14.54	307.30^ab^ ± 17.19	2.58 × 10^−7ab^ ± 2.44 × 10^−7^	315.88^ab^ ± 26.62	2.45 × 10^−7ab^ ± 2.24 × 10^−7^
HG	Yes	LT	2.06 × 10^8d^ ± 2.63 × 10^7^	113.44^bc^ ± 19.03	130.54^de^ ± 28.87	5.76 × 10^−7b^±4.13 × 10^−8^	94.45^de^ ± 4.71	4.52 × 10^−7b^±4.82 × 10^−8^
HTST	No	HT	2.20 × 10^9b^ ± 2.95 × 10^8^	114.87^bc^ ± 7.95	269.71^bc^ ± 14.47	1.25 × 10^−7ab^ ± 1.26 × 10^−8^	181.95^bc^ ± 8.66	8.36 × 10^−8ab^ ± 7.40 × 10^−9^
HGHTST	Yes	HT	1.65 × 10^9bc^ ± 4.28 × 10^8^	121.34^ac^ ± 14.90	181.65^ce^ ± 24.63	1.11 × 10^−7ab^ ± 1.41 × 10^−8^	120.05^ce^ ± 26.27	7.26 × 10^−8ab^ ± 7.03 × 10^−9^
UHT	No	UHT	3.15 × 10^9b^ ± 1.78 × 10^9^	117.78^ac^ ± 9.13	68.12^df^ ±15.07	2.85 × 10^−8a^ ± 1.32 × 10^−8^	98.02^de^ ± 10.93	4.10 × 10^−8a^ ± 2.16 × 10^−8^
HGUHT	Yes	UHT	1.10 × 10^9c^ ± 2.48 × 10^8^	146.86^d^ ± 17.78	38.91^f^ ± 9.20	3.30 × 10^−8a^±3.32 × 10^−9^	51.56^d^ ± 9.43	4.87 × 10^−8a^ ± 3.55 × 10^−9^

*Note*: Results are reported as mean ± SD. Different superscript letters denote differences between treatment groups (*p* < .05).

Abbreviations: EV, extracellular vesicle; HG, homogenization; HGHTST, homogenized high‐temperature short‐time; HGUHT, homogenized ultra‐high‐temperature; HTST, high‐temperature short‐time; LT, low‐ temperature; Reference, raw unprocessed milk; SD, standard deviation; UHT, ultra‐high‐temperature.

### 3.4 MiRNA and protein quantification

**FIGURE 4:**
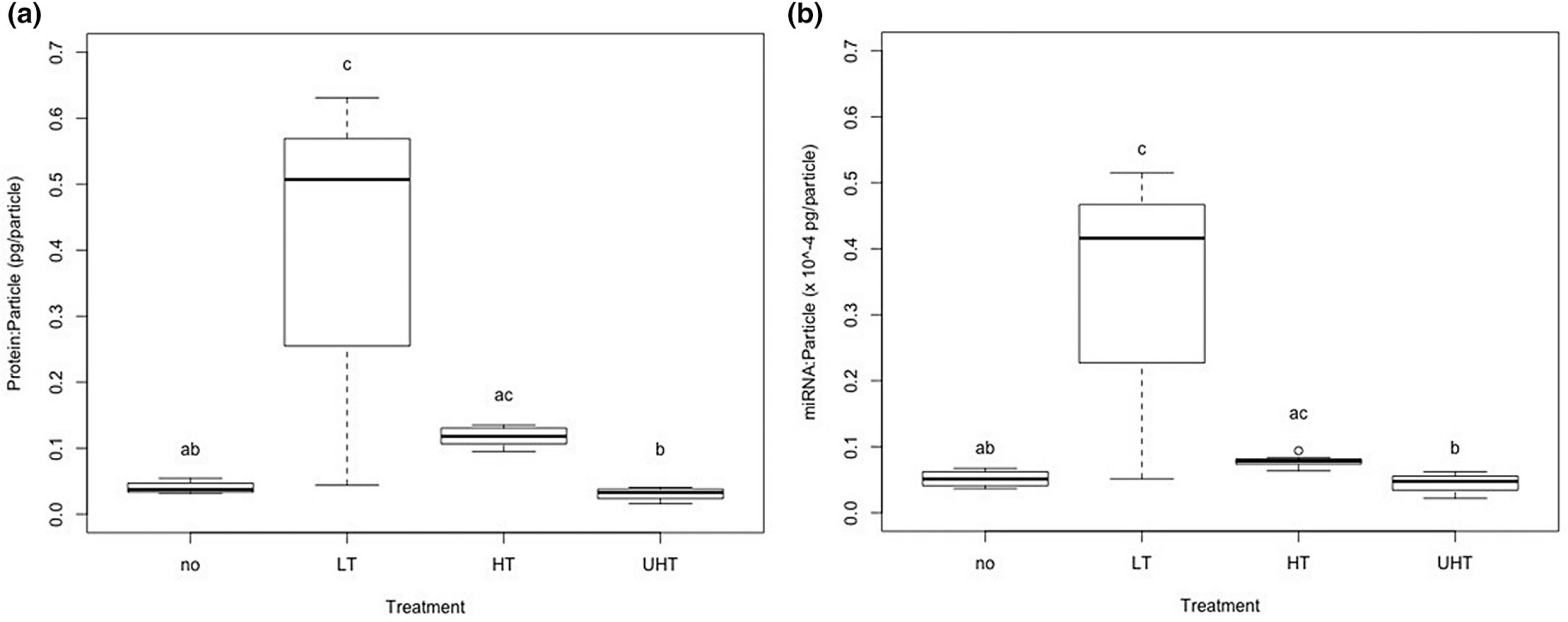
Boxplot of miRNA protein concentrations adjusted to total particle number from bovine milk extracellular vesicle (EV) isolations. (a) Low‐temperature **(**LT**)** treatment resulted in increased miRNA/particle concentration compared to the high‐temperature (HT) and ultra‐high‐temperature (UHT) groups (*p* < .05). (b) Low‐temperature (LT) treatment resulted in increased protein/particle concentration compared to high‐temperature (HT) and ultra‐high‐temperature (UHT) groups (*p* < .05). Medians were compared using Kruskal–Wallis (*n* = 28). Different superscript letters indicate significant differences.

The total miRNA value for the raw milk was 5.16 × 10^−8^ ± 1.34 × 10^−8^ ng/particle and data for each treatment group are summarized (Table 1). There was a ninefold increase (*p* = .05) in the average miRNA/particle concentration for EVs in the HG group compared to the reference. Independent of homogenization, HTST or UHT treatment of milk did not decrease the average miRNA/particle concentration relative to the raw milk (Figure 4a). However, the average miRNA/particle concentration was 25% lower (*p* < .01) in EVs from the HTST treatment group compared to LT, and 50% lower (*p* < .01) in EVs from the UHT‐treated milk compared to HTST‐treated milk (Figure 4a).

The total protein value for the raw milk was 4.02 × 10^−8^ (±1.01 × 10^−8^) μg/particle and data for each treatment group are summarized (Table 1). There was a 14‐fold increase (*p* = .04) in the average protein/particle concentration for EVs in the HG group compared to the reference. Independent of homogenization, HTST or UHT treatment of milk did not decrease the average protein/particle concentration relative to the raw milk (Figure 4b). However, the average protein/particle concentration was reduced by more than 75% (*p* < .01) in EVs for HTST and UHT treatments compared to LT (Figure 4b).

## 4 DISCUSSION

The results of this study clearly show that heat processing and homogenization independently and additively reduce the content of EVs in bovine milk. Lower‐temperature heat treatment selects for populations of EVs with higher miRNA and protein content compared to pasteurization at higher temperatures. Membrane proteins are integral to the structure, integrity, and resilience of EVs. Therefore, higher protein density may confer resilience of EVs in bovine milk to low‐temperature heat processing, but not to pasteurization. In addition to the presence of EVs in milk products and resilience to processing, EV bioactivity relies on relative bioavailability in consumers including resistance to digestion and passage through physiologic barriers. In combination with other studies on bioavailability (Khanam, 2023), the results of this study are important for conferring the potential health benefits of EVs to consumers of specific bovine milk‐derived nutrition products.

### 4.1 Extracellular vesicle characterization

The method utilized for the isolation of EVs from raw bovine milk was validated by quantitative description of the source of EVs, quantitative assessment of the EV preparation by the total protein concentration, global characterization showing at least three positive and at least one negative marker, and characterization of single vesicles in accordance with guidelines suggested from the Minimal Information for Studies of EVs (Théry et al., 2018). The mean concentration of EVs from raw bovine milk reported in this study was similar to results reported from semi‐skimmed commercial milk samples which also used a differential centrifugation protocol (Pieters et al., 2015). The mean protein concentration of EVs reported in this study was lower than in a report of bovine milk exosomes isolated from store‐bought skimmed milk using a commercial isolation kit (Carobolante et al., 2020). However, evidence suggests that the use of precipitating reagents may result in the coprecipitation of non‐EV proteins and impurities (Jan Van et al., 2014; Konoshenko et al., 2018; Lobb et al., 2015). The confirmation of at least one transmembrane or glycosylphosphatidylinositol‐anchored protein associated with plasma membrane and/or endosomes is required, and the presence of the non‐tissue‐specific tetraspanins CD63 and CD81 was confirmed in this study. The EVs isolated from raw bovine milk were positive for at least three known positive EV markers and negative for at least one known negative EV marker. Finally, the broad‐field and close‐up images obtained from SEM (diameters <200 nm) were consistent with the diameters of EVs (~130 nm) assessed by NTA in this study, and in agreement with findings reported in literature (Yamauchi et al., 2019) for raw bovine milk EVs (125 nm). Size of EVs isolated from raw bovine milk showed a unimodal distribution, and similar size distributions for bovine milk EVs have been previously evidenced in literature (Munagala et al., 2016; Somiya et al., 2018). Collectively, these observations suggest that the isolation method used was adequate for downstream analysis of EVs from raw milk.

### 4.2 Extracellular vesicle quantification

All processing treatments resulted in reduced concentration of EVs compared to raw milk. Any heat treatment resulted in a significant decrease in EV quantity. Previous literature also reported that UHT‐treated milk has a lower concentration of EVs in comparison to raw milk (Kleinjan et al., 2021). Homogenization also reduced the presence of EVs in bovine milk. Cavitation refers to the pressure variation in homogenizers which creates vapor bubbles that implode and produce shock waves that disrupt globules and nanostructures. Changes in EV concentration resulting from homogenization may thus be due to the shearing forces and high pressures inherent to this treatment (Howard et al., 2015). These notions are supported by evidence showing a decrease in human milk miRNA resulting from high‐pressure processing (Smyczynska et al., 2020), presumably due to the disruption of EV membranes. It is unknown whether processing affects subsets of EV populations uniformly, but it has been suggested that processing may induce the loss of large EVs specifically (Shome et al., 2021). Finally, it is plausible that heat treatment and homogenization pressure cause denaturation of EV proteins resulting in the loss of vesicular integrity.

In addition to quantifying the reduction in milk EVs or milk RNA by processing, some studies have shown that processing alters the structural composition or size of EVs intrinsic in the milk food matrix. Homogenization may alter the physicochemical arrangement of EVs (Michalski & Januel, 2006). EVs in raw milk are spherical with a clear lipid bilayer, and the morphology of EVs processed by homogenization and pasteurization was markedly different from that of raw milk EVs (Kleinjan et al., 2021). Additionally, casein micelles may be disrupted by high‐pressure homogenization (Fox et al., 2015) and adsorb at the EV interface in micellar form or as fragments (Michalski & Januel, 2006) altering the yield from isolation. In addition, homogenization shear and pipeline shear at higher pressures have been proposed to change the size of liposomes (Tai et al., 2019), which bear structural similarities to EVs. These studies suggest that EV composition may be altered by components of the bovine milk matrix during processing and further investigation warrants whether there is alteration in the bioactivity of EVs after processing.

### 4.3 MiRNA and protein content of EVs

The miRNA/particle concentration was higher in milk treated at low temperature than in raw milk. This finding may be due to the selective loss of specific EV subsets that have lower miRNA content. Other research has shown a reduction in the content of small RNA in pasteurized milk compared to raw milk (Kleinjan et al., 2021). Significant reductions in miRNA read counts were also identified in high heat‐treated milk (boiled, extended shelf‐life, and UHT) (Kirchner et al., 2016). Decreases in specific milk miRNA (miR‐29b and miR‐200c) have been reported after pasteurization, homogenization, and microwave heating, and it was proposed that these miRNAs were lost due to disruption of exosomal membranes (Howard et al., 2015). However, read counts of specific miRNA with putative targets related to immune response and growth were not different across six different milk treatments compared to raw whole milk (Shome et al., 2021). MiRNAs were not detectable in bovine milk‐based infant formulas (Leiferman et al., 2019). Since the majority of miRNA in milk is encapsulated in EVs (Baier et al., 2014; Rani et al., 2017; Zhou et al., 2012), the resilience of milk miRNA is contingent on the membrane integrity of EVs. As this study showed that low‐temperature processing preserved EVs with the highest content of miRNA, continued research might focus on the resilience of specific populations of EVs (e.g., small and large) that contain bioactive miRNA.

Analogous to the findings for miRNA, only low‐temperature heat processing resulted in increased protein/particle concentration in EVs. Low‐temperature heating, but not pasteurization, may impart the selective loss of specific EV subsets that have lower protein content. In this study, larger EVs were apparently more sensitive to loss than small EVs when heated at low temperatures. Small EVs have a higher ratio of protein in the membrane as compared with the cargo (Zendrini et al., 2022) which presumably confers protection to the vesicles. This may have functional consequences for consumer milk products in which specific bioactive proteins are differentially enriched in large and small EV populations (Lischnig et al., 2022).

As in this study, previous evidence showed no additional decrease in total protein content of EVs from bovine milk with homogenization of heat‐treated samples (Kleinjan et al., 2021). Similar to the present study, a ratio of 2 × 10^−8^ μg protein/particle has been reported in commercially available semi‐skimmed milk (Pieters et al., 2015). Another study reported a ratio of 4.17 × 10^−9^ μg protein/particle in EVs from store‐bought whole bovine milk which was lower, but still within one order of magnitude and in agreement with our findings relative to raw milk (Somiya et al., 2018). Collectively, the results from this study and others (Benmoussa et al., 2016; Izumi et al., 2012; Zhou et al., 2012) suggest that the content of miRNA in EVs may depend on the density of structural protein present that protects the integrity of the vesicle membrane.

### 4.4 Strengths and limitations

This study has several notable strengths. Ninety percent of U.S. dairy cows are Holstein, but other dairy‐producing breeds include Jerseys and crossbreeds (HAUSA, 2020; USDA, 2020). Importantly, the milk utilized in this research was a composite of two batches obtained from a farm typically supplied to a dairy processing plant. The results obtained are representative of EVs that would be present typically in commercially available milk because the present study utilized reference and processed milk treatment groups from the same batches. This study design consideration also minimized a number of potential confounders since the content and composition of EVs in bovine milk may vary according to the season, cattle's breed, and diet (Özdemir, 2020). This study was primarily descriptive of EV content and did not explore how functionality of EVs may be impacted by processing. Without acidification of the bovine milk sample, centrifugation at 100,000 *g* likely resulted in the loss of some EVs before final pelleting (at 130,000 *g*) and incomplete removal of casein micelles. EVs isolated from raw milk, but not from the other experimental groups were characterized using guidance from the MISEV.

## 5 CONCLUSION

Homogenization and heat treatments independently and additively result in the loss of EVs from bovine milk. Low‐temperature heat treatment of bovine milk selects for populations of EVs with higher miRNA and protein content. Higher protein content confers resistance to the apparent degradation of EVs in bovine milk by HTST processing, but not by the temperatures used in UHT treatment. This study contributes foundational knowledge on the effects of processing on raw bovine milk EVs. This research may inform future studies on the preservation of EVs in bovine milk‐based products which could positively impact health outcomes in consumers.

## AUTHOR CONTRIBUTIONS

**Anna p. Colella:** Data curation (lead); formal analysis (equal); investigation (equal); methodology (equal); validation (equal); visualization (lead); writing – original draft (lead). **Anuradha Prakash:** Conceptualization (supporting); funding acquisition (supporting); investigation (equal); methodology (equal); project administration (supporting); resources (supporting); supervision (supporting); validation (equal); writing – review and editing (supporting). **JOHN J. MIKLAVCIC:** Conceptualization (lead); data curation (supporting); formal analysis (equal); funding acquisition (lead); investigation (equal); methodology (equal); project administration (lead); resources (lead); software (lead); supervision (lead); validation (equal); visualization (supporting); writing – original draft (supporting); writing – review and editing (lead).

## FUNDING INFORMATION

The study is funded by the USDA (2020‐67018‐33315).

## Supporting information


Appendix S1

## Data Availability

Per the funding application Data Management Plan, the data are not publicly available but can be requested from the corresponding author.
