# Supplementary material for: Homogenization and thermal processing reduce the concentration of extracellular vesicles in bovine milk
Source: Food Sci Nutr. 2023 Oct 8;12(1):131–40. doi: 10.1002/fsn3.3749 (PMC10804120; doi:10.1002/fsn3.3749)
Supplement: Supplementary file 1 — Appendix S1 [file FSN3-12-131-s001.docx]

| Batch | Fat (%) | Protein (%) | Lactose (%) | Total Solids (%) | Solids-not-fat (%) |
| --- | --- | --- | --- | --- | --- |
| M1 (n=9) | 3.64 ± 0.01 | 3.14 ± 0.01 | 4.62 ± 0.01 | 12.50 ± 0.02 | 8.82 ± 0.01 |
| M2 (n=9) | 3.70 ± 0.01 | 3.16 ± 0.00 | 4.55 ± 0.01 | 12.53 ± 0.01 | 8.79 ± 0.01 |
| Average | 3.67 ± 0.04 | 3.15 ± 0.02 | 4.58 ± 0.04 | 12.51 ± 0.02 | 8.81 ± 0.02 |

**Supplemental Table 1.** Physical properties summary of raw bovine milk.


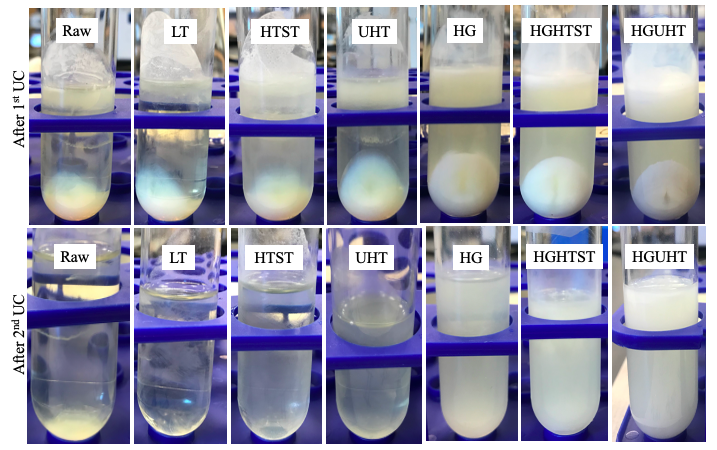


Supplemental Figure 1 Differences in turbidity across treatments after first and second UC spins for the EV isolation using a differential centrifugation protocol.


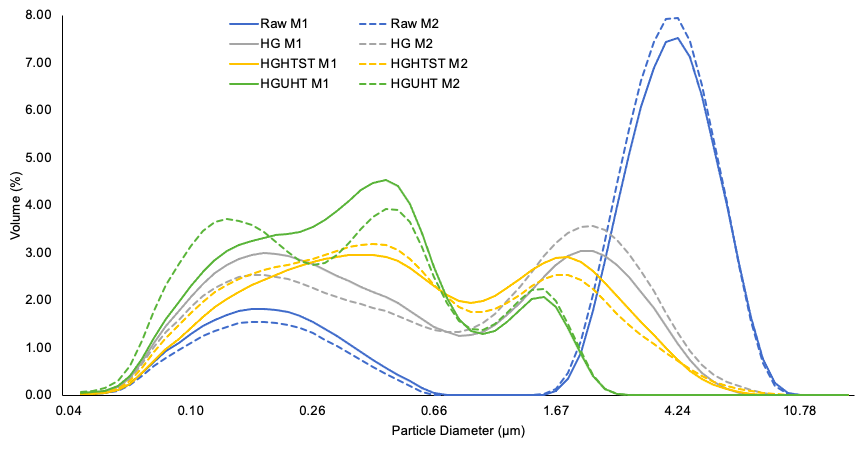


Supplemental Figure 2 Size distribution for homogenization validation (*n* = 24). Raw milk displays the largest volume of particles with a peak in the volume % of particles with a diameter of 4.24 µm, while all homogenized milk group (HG, HGHTST, HGUHT) resulted in lower non-uniform peaks and smaller particle diameters. M1 = batch 1, M2 = batch 2.


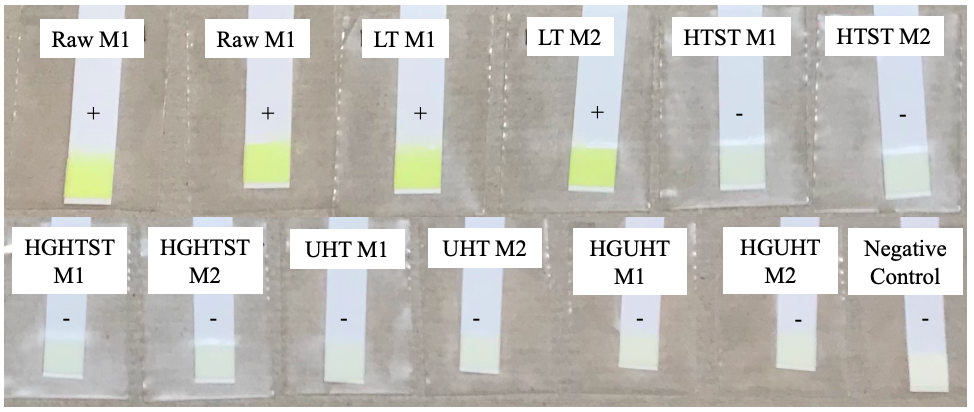


Supplemental Figure 3 ALP Test Results. ALP-inactivated milk samples (HTST, HGHTST, UHT, HGUHT) appear in white, while active ALP samples (raw, LT) appear in yellow.

**
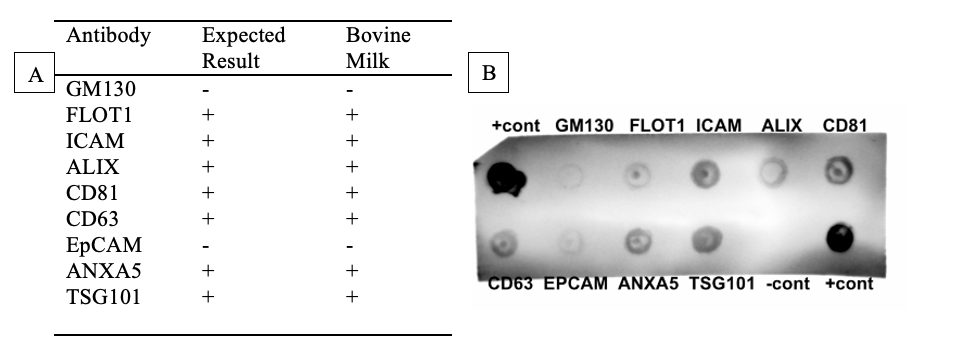
**

**Supplemental Figure 4.** Global characterization for EV markers. Expected and obtained results for the semiquantitative immunoblot of EVs from raw bovine milk (A). Semi-quantitative immunoblot of raw bovine milk EVs. +cont and -cont represents positive and negative controls, respectively. EpCAM and GM130 are markers of cellular contamination (B). TSG101, FLOT1, ANXA5, ALIX, CD63, CD81, ICAM are positive markers of EVs.


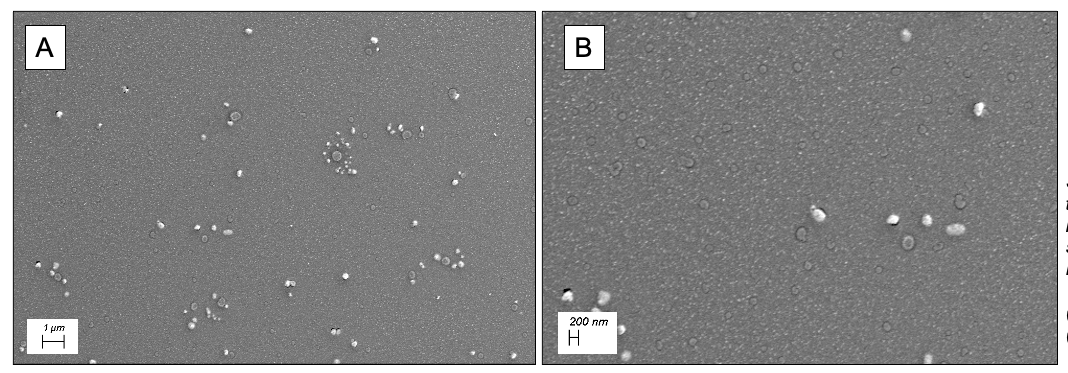


**Supplemental Figure 5.** Images of raw bovine milk extracellular vesicles (EVs) obtained by scanning electron microscopy (SEM). (A) Broad-field image, 1:3,500 dilution, 1 µm scale. Electron high tension = 10.00 kV, signal A = SE2, aperture size = 30.00um, working distance = 7.3 mm, magnification = 4.74 K X, specimen height = 2mm. (B) Close-up image, 1:3,500 dilution, 200nm scale. Electron high tension = 10.00 kV, signal A = SE2, aperture Size = 30.00um, working distance = 7.3 mm, Mag = 4.74 K X, specimen height = 2mm.
